# Developing a core outcome set for clinical trials of traditional Chinese medicine for rheumatoid arthritis

**DOI:** 10.3389/fmed.2025.1690963

**Published:** 2025-12-08

**Authors:** Bingbing Yu, Mengge Song, Jingdong Yang, Xiaopo Tang, Congmin Xia, Junjie Zhang, Xue qi Zhao, Quan Jiang, Man Han

**Affiliations:** 1 Division of Rheumatology, Guang’anmen Hospital, China Academy of Chinese Medical Sciences, Beijing, China; 2 School of Optical-Electrical and Computer Engineering, University of Shanghai for Science and Technology, Shanghai, China

**Keywords:** rheumatoid arthritis, core outcome set, traditional Chinese medicine, Delphi survey, semi-structured interview

## Abstract

**Objective:**

We developed a consensus-based core outcome set (COS) for Traditional Chinese Medicine clinical trials in rheumatoid arthritis (COS-TCM-RA) that to addresses heterogeneity in outcome measurement and reporting across studies.

**Methods:**

We performed a comprehensive systematic review to establish a repository of clinical outcome set for TCM interventions in RA management. Then, a semi-structured interview was conducted to identify additional important outcomes from the patient’s perspective. This was followed by an online three-round Delphi survey conducted with professionals to prioritize and refine clinically relevant outcomes from a previously developed longlist. Core entries were finalized based on three Delphi surveys and one expert consensus meeting.

**Results:**

The systematic review screened 2,959 records and included 69 eligible studies. From these, a comprehensive list of reported outcomes was extracted and standardized, which after standardization, yielded a final list of 52 unique outcomes for the preliminary consensus process. After semi-structured interviews, three Delphi survey rounds and one consensus meeting, the most important outcomes were determined for COS of Traditional Chinese Medicine for RA (COS-TCM-RA), including physician assessment of global status, Clinical laboratory tests, radiographic assessment, Signs and symptoms, quality of life and security incident.

**Conclusion:**

This study developed the first COS-TCM-RA, comprising 11 indicators across five domains: overall disease evaluation, physical and chemical indicators, quality of life, TCM syndromes, and adverse events. By integrating internationally recognized RA assessment tools with patient-reported outcomes and TCM-specific measures, the COS-TCM-RA provides a standardized, multi-dimensional framework to enhance trial quality, improve comparability, and support the integration and internationalization of TCM in evidence-based medicine.

## 1 Introduction

Rheumatoid arthritis (RA) is a chronic, systemic autoimmune inflammatory disease that primarily affects the joints and periarticular soft tissues (1), causing progressive cartilage and bone damage and disability (2), with a global prevalence of 0.5 to 1%. Traditional Chinese Medicine (TCM), grounded in a unique theoretical framework and practiced for over 2,000 years, is widely applied in China for the clinical management of RA (3), and clinical evidence demonstrates its therapeutic potential (4). It adopts a holistic perspective and individualized therapeutic approaches, including herbal medicine, acupuncture, and moxibustion (4). Currently, integrated treatment strategies combining TCM with Western medicine are commonly used in clinical practice, with many patients selecting TCM as their primary or adjunctive treatment modality. An increasing body of clinical research suggests that these interventions may exert therapeutic effects through mechanisms such as modulation of the immune network and inhibition of pro-inflammatory cytokines (5, 6). However, standardized methods specifically designed to evaluate the efficacy of TCM for RA are currently lacking. Moreover, significant heterogeneity in outcome measures across clinical trials impedes consistent efficacy assessment, limiting evidence synthesis and comparability among studies.

COS represent the minimum outcomes that should be measured and reported in clinical trials in a specific area of healthcare (7). At present, COS are increasingly being developed to identify outcomes important to decision makers, improve outcome reporting, and standardize definitions and measures. The internationally recognised RA-OMERACT provides a foundation for standardised assessment of disease activity. However, existing international standards fail to encompass patients health-related quality of life, including subjective experiences across physical, psychological, cognitive and social dimensions, nor do they sufficiently integrate safety monitoring into efficacy assessments (8). At the patient-reported outcome level, there is a lack of assessment tools suitable for specific cultural contexts. By systematically incorporating TCM syndromes and safety indicators, and employing localised reporting tools suitable for Chinese patient populations. Consequently, this study aims to develop a core set of outcomes specifically for TCM treatment of RA (COS-TCM-RA). The establishment of a COS for RA in TCM clinical research (COS-TCM-RA) offers a promising solution to address the heterogeneity of outcome measures in current TCM trials in RA. Based on the COS-TCM-RA technical specifications, our research team convened a panel of experts from the Rheumatology Branch of the Chinese Society of Traditional Chinese Medicine to develop consensus on standardized endpoints for TCM clinical trials targeting RA. This standardized COSwill enhance comparability across studies, improve clinical trial quality and efficiency, and facilitate objective evaluation of TCM therapeutic effects. Furthermore, implementing COS in TCM research will promote standardization and internationalization, ultimately advancing TCM’s global recognition in evidence-based medicine.

## 2 Methods

This study was part of the RA Chinese Medicine Clinical Research Core Indicator Set project, which was registered in the COMET database.^1^ Permission for this study was obtained from the Ethics Committee of Guang’anmen Hospital, China Academy of Traditional Chinese Medicine (2022-170-KY).

### 2.1 Design: the protocol involved five steps

Step 1. We conducted a comprehensive systematic literature review to identify, evaluate, and synthesize the reported outcomes in clinical trials of Traditional Chinese Medicine (TCM) for RA.
Step 2. A semi-structured interview was conducted to explore the impact of the disease on patients’ daily living before and after treatment, as well as the improvements resulting from Traditional Chinese Medicine.
Step 3. The first two rounds Delphi survey were conducted to prioritize and condense the outcomes.
Step 4. A consensus meeting was held to further deliberate on the RA-TCM-COS.
Step 5. The final round of Delphi surveys to develop the RA-TCM-COS.

**Figure 1:**
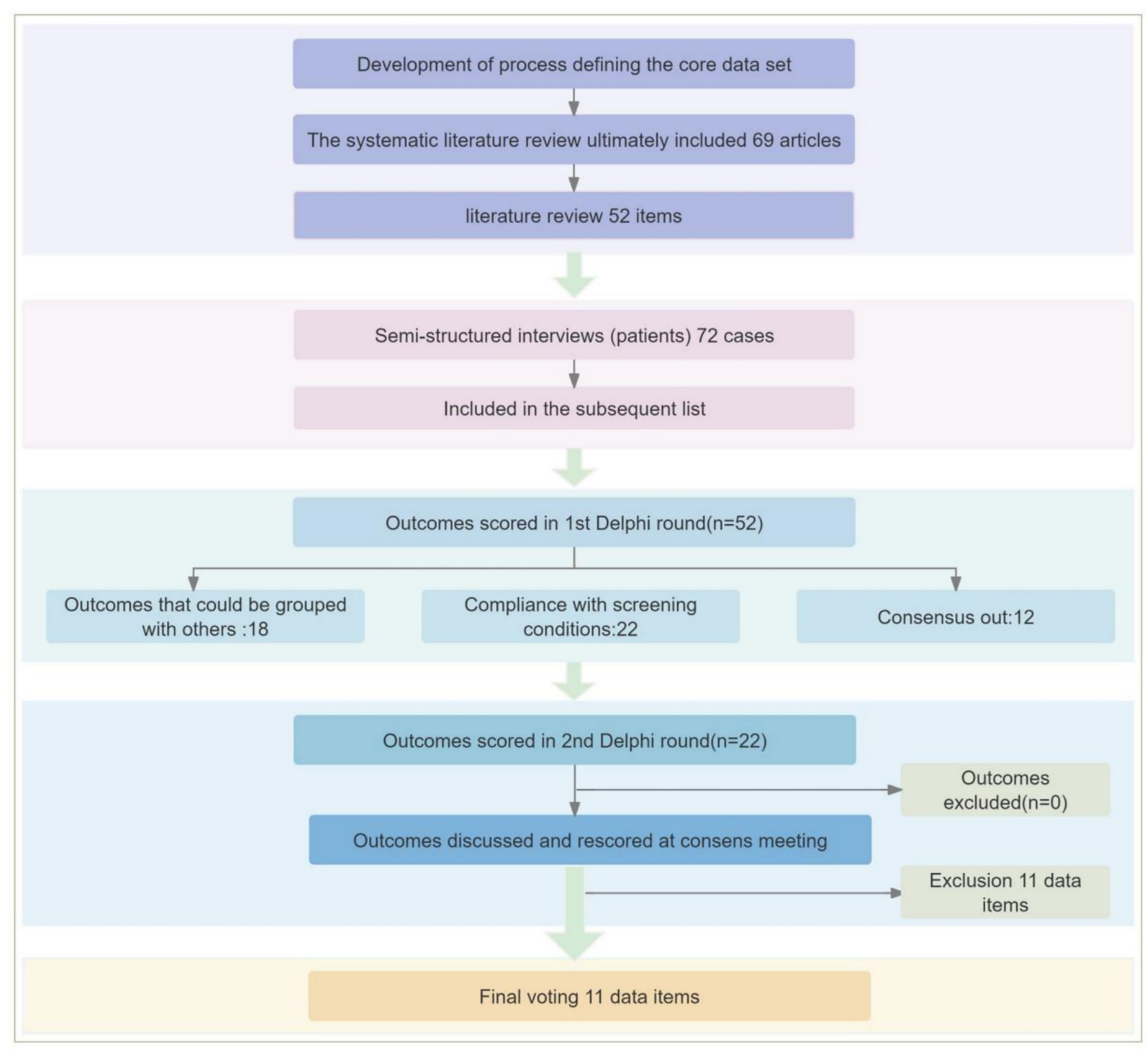
Flow chart of the development and consensus process for the COS.

The study was conducted according to the Core Outcome Measures in Effectiveness Trials (COMET) Handbook: Version 1.0, a guideline for COS development (9). The methodological design of this study aligns with the consensus standards established by the COS initiative. It adopts the minimum standards for development proposed by COS-STAD as the primary framework for programme construction, clearly defining the scope of research (10), stakeholder composition, and consensus-building process (Figure 1).

### 2.2 The scope of the COS

The COS-TCM-RA is developed to be applied in clinical studies evaluating Traditional Chinese Medicine interventions for adults with rheumatoid arthritis. Its specific scope is defined as follows:

(1) Health Condition Rheumatoid arthritis, as defined by internationally accepted classification criteria (ACR/EULAR 2010 criteria).
(2) Target Population: Adult patients (≥18 years) diagnosed with RA, who had either gone through Traditional Chinese Medicine treatment previously or were currently in the course of receiving it.
(3) Interventions: All therapeutic modalities of Traditional Chinese Medicine used in the management of RA, including but not limited to Chinese herbal medicine, acupuncture, moxibustion, and Tuina. This COS is also intended for use in studies evaluating integrated TCM and Western medicine treatment strategies.
(4) Settings: The primary setting for application is clinical trials. It may also be used to standardize outcome assessment in routine clinical practice to evaluate the effectiveness of TCM for RA.

### 2.3 Step 1: systematic literature review

We systematically reviewed recent (2019–2024) clinical studies on TCM for RA to identify current evidence on treatment outcomes. We searched the following Chinese and English databases using MeSH terms and keywords: We searched three English databases (PubMed, The Cochrane Library, and Embase) and four Chinese databases (China National Knowledge Infrastructure [CNKI], SinoMed, China Science and Technology Journal Database and Wanfang Database). In order to collect comprehensive outcomes, we also searched two clinical trial registries^2^ to retrieve any outcomes used in clinical trials.

#### 2.3.1 Inclusion criteria

(1) Study Types: randomized controlled trials, non-randomized controlled trials, case series, case–control, cohort studies, and systematic reviews evaluating TCM for RA.
(2) Study population: Adult patients (≥18 years) with a confirmed diagnosis of rheumatoid arthritis based on internationally accepted ACR/EULAR 2010 criteria.
(3) Interventions: Any TCM-related monotherapy or combination therapy, including but not limited to: Chinese herbal medicine, acupuncture, moxibustion, tuina, and medicated baths.
(4) Outcome measures: Studies that reported at least one clinical outcome measure.

#### 2.3.2 Exclusion criteria

(1) Non-clinical studies (basic science, *in vitro* experiments).
(2) Review articles, commentaries, or editorials without original data.
(3) Degree theses and conference papers.
(4) Duplicate publications.

#### 2.3.3 Data extraction

Two reviewers (SMG and YBB) independently extracted the data using a pre-designed data extraction form. Disagreements were resolved by discussion or consulting a third researcher (ZXQ). The extracted information included:

(1) Study characteristics: First author, publication year, journal, country, study design, sample size, funding sources.
(2) Participant characteristics: Age, gender, disease duration, RA diagnostic criteria used, TCM syndrome pattern.
(3) Intervention details: Type of TCM therapy, dosage, frequency, treatment duration, and details of any control interventions.
(4) Outcome measures: For every outcome reported in a study, we extracted the following:

Outcome domain and specific name (Disease Activity: DAS28; Pain: Visual Analogue Scale score; Physical Function: Health Assessment Questionnaire).

Measurement instrument or tool (DAS28-CRP, VAS, HAQ-Disability Index, Szkudlarek M Standard for ultrasound synovitis grading).

Specific metric used (change from baseline, endpoint value, proportion of patients achieving remission).

**Figure 2:**
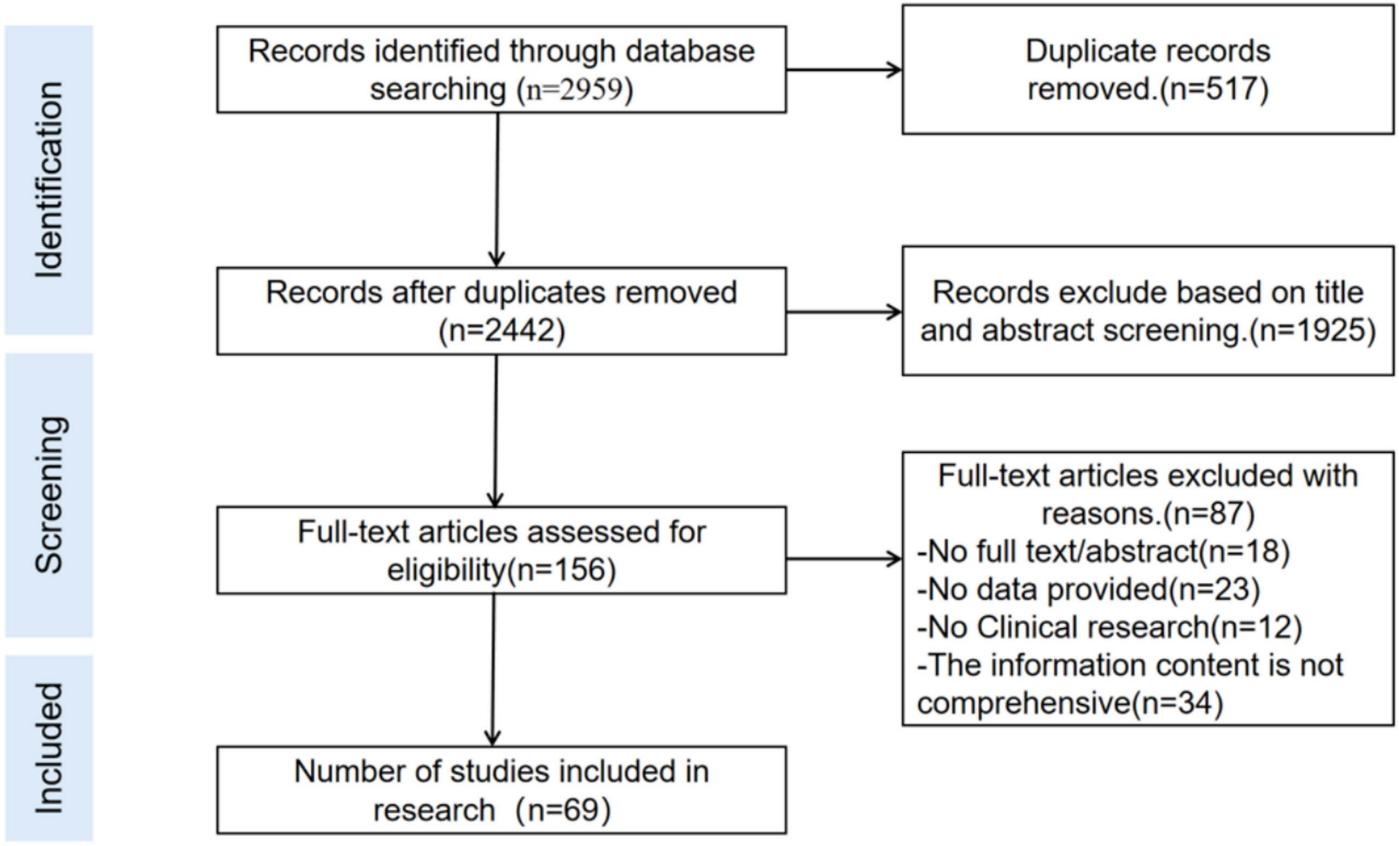
Flowchart of article identification and selection.

Time points of assessment (baseline, week 4, week 12, week 24) (see Figure 2).

### 2.4 Step 2: semi-structured interview

#### 2.4.1 Participant recruitment and sample size

Outcome selection was guided by a multi-stakeholder consensus process. Open-ended interviews served as the foundational step for subsequent quantitative prioritization. The sample size was determined by integrating purposive stratified sampling with the principle of thematic saturation. Participants were recruited through purposive stratified sampling based on the following variables: (1) gender (male, female); (2) age group (18–40, 41–65, >65 years); (3) disease stage, defined as early (<2 years), mid-term (2–5 years), or late (>5 years); (4) disease activity, classified by DAS28-CRP score as low (<3.2), moderate (3.2–5.1), or high (>5.1); (5) educational level (primary school or below, junior high school, senior high school, or university and above). (Eligible patients were those who had experienced RA-related symptoms and had either previously undergone or were currently receiving TCM treatment, thereby possessing firsthand knowledge of treatment outcomes). Participants rated their experiences on a 9-point Likert scale, with higher scores representing greater disease-related distress at baseline or more substantial improvement following treatment. This study systematically gathered and analyzed patient-reported concerns and priorities regarding treatment efficacy. During qualitative data analysis, we employed Braun and Clarke’s reflexive thematic analysis (RTA) to construct a coding framework: two researchers independently conducted open coding of interview transcripts, refining initial concepts into consensus-based thematic domains through multiple rounds of comparative discussion. Finally, outcomes were ranked based on their scores to facilitate the analysis and comparison of perspectives across different patient subgroups.

#### 2.4.2 Data collection

The questions asked during the interview revolved around Chinese medicine therapies for RA. After receiving clarification on the study purpose and the definition of outcomes, the patients were asked to suggest relevant outcomes based on their experience with RA, potentially important outcomes following Chinese medicine treatment and their reasoning based on these choices judgments. The outline items are as follows:

(1) When was your RA diagnosed?
(2) What are the three most troubling symptoms before treatment?
(3) What are the three conditions that show the most significant degree of improvement after treatment?
(4) Score these in turn.

Narrative data were indexed and charted to produce a thematic framework. These patient centered outcomes were reviewed and added to the candidate outcome list. Table 1 presents the percentage of patients indicating the degree of distress prior to treatment and the degree of improvement following treatment, along with the corresponding mean scores (rating 7 or higher on the 9-point Likert scale).

**Table 1:** Characteristics of the final core outcome indicators and expert consensus results.

Outcome indicator	Inconclusive	Disagreement	Consent	Percentage agreement	Reach a consensus
Overall disease evaluation (subjective and objective)					
DAS28	0	0	38	100.00	√
PRO (CPRI-RA)	3	0	35	92.11	√
Physical and chemical indicators					
CRP	0	0	38	100.00	√
ESR	0	0	38	100.00	√
Ultrasound: Szkudlarek M Standard	6	1	31	81.58	√
Quality of life					
HAQ-DI	0	0	38	100.00	√
TCM-syndromes					
Syndrome effectiveness evaluation scale	2	0	36	94.74	√
AEs					
Adverse event rate	1	0	37	97.37	√
Liver function	1	0	37	97.37	√
Kidney function	1	0	37	97.37	√
Routine blood test	2	0	36	94.74	√

DAS28, Disease Activity Score in 28 joints; CPRI-RA, Chinese patient-reported activity index with RA; CRP, C-reactive protein; ESR, Erythrocyte sedimentation rate; HAQ-DI, Health Assessment Questionnaire-Disability Index; AEs, adverse events.

### 2.5 Step 3: two-round Delphi survey

#### 2.5.1 First round of the Delphi survey

(1) Delphi survey panel assembly

Following principles of representativeness and clinical expertise, we recruited rheumatology specialists practicing Traditional Chinese Medicine (TCM), integrated TCM-Western medicine and evidence-based medicine methodologists. The expert panel comprised rheumatologists from 86 clinical centers across all seven geographic regions of China (North, Northeast, East, Central, South, Southwest, and Northwest), representing 29 provincial-level administrative divisions (provinces, municipalities, and autonomous regions). All experts were selected based on their high professional qualifications, with 5 ~ 40 years of clinical experience in the field of rheumatology. Their extensive expertise and experience further confirm their authority in the field, ensuring the credibility and relevance of the Delphi method in establishing the COS indicators. Detailed demographic characteristics of the participants can be found in Appendix 2.

(2) Rating and refining outcomes

For the first Delphi round, participants were provided with an electronic questionnaire containing outcome measures identified through systematic literature review and Semi-structured interview.

To evaluate the outcomes’ importance, we used a 9-point Likert scale, where 1, 2, and 3 meant “unimportant” 4, 5, and 6 meant “important, but not essential”; and 7, 8, and 9 meant “essential” (11). If participants were uncertain about the importance of an outcome indicator, they could select “Not sure” as a response option.

Outcomes that received consensus in the first round, defined as being rated as critical’ by at least 70% of participants and not important’ by fewer than 15%, were retained as potential core outcomes. Conversely, outcomes rated as “not important” by 70% of participants and “critical” by fewer than 15% were considered for exclusion from the COS. To adhere to the COS principle of a minimal set and to keep the subsequent questionnaire manageable, the core steering group agreed to retain no more than four outcomes from each domain for the second round. When a domain yielded more than four eligible outcomes, the core experts re-evaluated their clinical relevance and consensus ratings, and selected four representative indicators to advance to the second round.

In the first round, the questionnaire was sent to experts, who were asked to complete the survey within 2 weeks. A reminder email was sent at the end of the first week. After 2 weeks, we performed a statistical analysis of the first-round results (12). These results included: (a) general content of establishment of the COS for clinical studies: number of questionnaires sent, number of questionnaires recovered, number of participants completed, and proportion of questionnaires completed; (b) scoring content: the scoring results and distribution of each outcome (including the mean, minimum, and maximum values of scores and the consensus of different scores). Outcomes with an average score of 7 or above were ranked from high to low. After the first round, we provided all participants with aggregated statistics. Each expert could view the summary results via the electronic questionnaire interface. The full set of scores from the 117 experts is available in Appendix 1.

#### 2.5.2 Second round of the Delphi survey

(1) Delphi survey panel assembly

The expert panel for this second round of the Delphi survey comprised 40 specialists, all holding the title of Associate Chief Physician or above (the vast majority being Chief Physicians). They were drawn from 31 high-calibre hospitals across 19 provinces, municipalities, and autonomous regions nationwide. With the goal of further refining and validating the core indicator set for TCM-COS-RA. Detailed demographic characteristics of the participants can be found in Appendix 2.

(2) The outcomes were further refined through the second Delphi round

All indicators that met the pre-specified consensus criteria for importance were carried forward to the consensus meeting for comprehensive deliberation, as the survey results alone were insufficient to reduce the list to a minimal core set.

### 2.6 Step 4: consensus meeting

The consensus panel consisted of 10 nationally recognized rheumatology experts with all holding leadership positions as standing committee members or above. Drawn from 7 provinces/municipalities across China, all members held chief physician titles with ≥25 years of clinical experience in either TCM or integrated TCM-Western Medicine practice, and had completed all Delphi survey rounds prior to the consensus meeting.

The expert panel conducted a hybrid (on-site/web-based) consensus meeting to: (1) clarify the COS framework, and (2) review the two-round Delphi survey results prior to final voting.

### 2.7 Step 5: final round of Delphi survey

The final round was conducted with a panel of 38 experts to finalize the outcome list. To ensure continuity and build upon prior consensus, the panel included 31 experts (81.6%) who had participated in all three Delphi rounds. A consensus exceeding 70% was reached for the final 11 item COS. The scoring data for the final round are detailed in Appendix 1.

### 2.8 Statistical analysis

Statistical analyses were primarily descriptive and followed pre-specified consensus rules. Continuous variables were summarized as median using medians or means ± standard deviations, and categorical variables were presented as counts and percentages. The Delphi survey used a 1–9 importance scale (1–3 = not important, 4–6 = important but not critical, 7–9 = critical). For each item, summary statistics (median, IQR, and the proportions of ratings in the 7–9 and 1–3 bands) were reported overall and by stakeholder strata (patients, clinicians, methodologists). Between rounds, participants received feedback of the prior round’s summaries to facilitate convergence. Consensus thresholds were pre-specified: items were included if ≥70% of ratings were in the 7–9 band and <15% were in the 1–3 band; excluded if the converse applied; all others were classified as undetermined and carried forward to the next round or discussed at the consensus meeting (this threshold aligns with conventions used in OMERACT and related consensus programs). For each round we recorded and reported the effective sample size, composition, and attrition. Sensitivity analyses were performed for borderline items (varying thresholds by ±5% or re-checking using overall-only summaries) to examine robustness. “Not sure” responses were excluded from denominators and presented descriptively only. At the consensus meeting, undetermined items were discussed under a structured agenda and decided by anonymous voting, applying the same inclusion threshold as in the Delphi.

#### 2.8.1 Consensus criteria

The first round of the Delphi survey employed a unified consensus criterion: indicators receiving scores of 7–9 from ≥70% of experts and scores of 1–3 from <15% were retained as high-importance indicators; those receiving scores of 1–3 from ≥70% of experts and scores of 7–9 from <15% were excluded by consensus; all others were classified as “undetermined” and advanced to the subsequent round of deliberation. The second Delphi survey and consensus meeting also adhered to this standard.

## 3 Results

The systematic review identified 2,959 records, from which 69 studies met the eligibility criteria. After extracting all reported outcome measures and undergoing a standardization process to merge synonyms and remove duplicates, a final list of 52 unique outcome items was generated. These outcomes were categorized into seven outcome domains: overall disease evaluation; signs and symptoms; clinical laboratory; radiographic assessment; Traditional Chinese Medicine syndrome; quality of life and security incidents. Subsequently, Seventy-two consecutively recruited RA patients completed semi-structured interviews. Quantitative analysis revealed the following symptom severity hierarchy: (1) joint pain, (2) disease diagnostic criteria, (3) disease global assessment, (4) fatigue, (5) joint swelling, (6) elevated inflammatory markers and; (7) Health-related quality of life (HRQoL). Post-treatment clinical assessments demonstrated improvement sequencing: (1) disease global assessment; (2) joint pain; (3) joint swelling followed by disease diagnostic criteria, laboratory values, fatigue, and muscle pain. Consequently, the patient group identified joint pain, disease global assessment, and joint swelling as highly important outcomes. These patient-prioritized outcomes were incorporated into the candidate list for the subsequent Delphi process.

Then, in the Delphi questionnaire, these indicators derived from patient interviews were specifically annotated with their patient origin and high priority ranking. This provided crucial contextual information for expert scoring, guiding them to fully consider the value of patient experience when weighing the importance of indicators. In the first Delphi round, all 117 invited experts (100% response rate) completed the survey. Following the pre-defined consensus criteria, 22 indicators were retained for the second round. Forty-two experts were invited to the second round, with 40 responses received (95.2% response rate). However, the results still contained a substantial number of high-priority outcomes, preventing the derivation of a minimal core set. Therefore, a consensus meeting with 10 experts was held, after which a third Delphi round was initiated. This final round employed a continuous sampling approach, involving 38 experts, 31 (81.6%) of whom had participated in all three rounds (Table 2).

**Table 2:** Patients’ distress after illness and improvement after treatment outcomes by semi-structured interviews.

Outline items	Agreement of distress after illness (%)	Average score for the degree of distress experienced after illness	Agreement of improvement after treatment	Average score for the degree of improvement following treatment
1. Disease global assessment	51.39	6.18	26.39	4.67
2. Joint pain	52.78	6.26	27.78	4.49
joint swelling	44.45	5.75	27.78	4.26
3. Duration of morning stiffness	331.94	4.82	19.44	3.61
4. Joint warmth	29.16	4.57	20.84	3.46
5. Muscle soreness	31.94	5.22	22.22	3.89
6. Inflammation-related markers such as ESR and CRP	41.67	5.67	18.06	3.81
7. RF, anti-CCP and other disease diagnostic markers	51.39	6.21	22.22	4.21
8. Results of wrist joint ultrasound, X-ray and other examinations	29.16	5.03	23.61	4.03
9. Upper limb mobility	38.89	5.38	19.44	3.57
10. Lower limb mobility	37.5	5.04	20.84	3.68
11. Fatigued state	47.23	5.83	25.00	3.92
12. Appetite	27.79	4.28	20.83	3.4
13. Emotional state	37.5	5.21	22.22	3.71
14. Illness has led to difficulties in daily life, work, and studies	37.5	5.49	19.45	3.78
15. Traditional Chinese Medicine syndromes (aversion to wind and chill, soreness and weakness in the lower back and knees, etc.)	443.06	6	23.61	3.96
16. Long-term prognosis (bone destruction, joint deformity)	29.16	4.61	22.22	3.74
17. Security incident(Adverse reactions following treatment, such as gastrointestinal discomfort)	15.28	3.83	–	–
18. Economic evaluation (treatment costs, cost–benefit ratio)	37.5	5.21	–	–

## 4 Discussion

This study developed the first COS for clinical trials of TCM in rheumatoid arthritis (RA). The development process has been registered with the COMET database. The COS-TCM-RA developed in this study integrates TCM-specific characteristics, internationally validated metrics and patient-reported outcomes, directly addressing the heterogeneity, and limited comparability of outcomes in existing TCM-RA clinical research. This provides a unified and authoritative outcome indicator framework for future TCM-RA clinical trials (13).

Conducted by a team of 117 authoritative experts from 86 hospitals nationwide, the research adopted rigorous methodologies including systematic review, patient interviews, three-round Delphi surveys, and expert consensus meetings, and ultimately established five indicator domains: overall disease evaluation, physical and chemical indicators, quality of life, TCM syndromes, and adverse events. The Disease Activity Score in 28 joints (DAS28), endorsed by the ACR and EULAR (14), remains the most widely used measure of RA activity. When paired with the Chinese-developed CPRI-RA (15), it allows comprehensive evaluation of joint function and patient experience. Notably, early detection and quantitative monitoring of both inflammation and structural damage are critical in RA management and in evaluating the effectiveness of interventions (16, 17). Laboratory markers such as the erythrocyte sedimentation rate and C-reactive protein are well-established indicators of systemic inflammation. Musculoskeletal ultrasound (MSUS) (18, 19) provides a standardized way to detect synovial inflammation, joint effusion, power Doppler signals and bone erosions using the Szkudlarek 0–3 semi-quantitative grading system (20). Beyond inflammation and joint destruction, RA is a chronic disabling disease that imposes long-term limitations on dressing, walking, hygiene and independent living (21). TCM-COS-RA includes the Health Assessment Questionnaire–Disability Index (HAQ-DI) as the primary tool for assessing physical function (21). Currently, the evaluation of TCM efficacy is constrained by the lack of quantitative methods for TCM-specific syndrome indicators, hindering comprehensive and objective assessments. Syndrome patterns, as the core indicators for TCM efficacy evaluation, are of particular importance for standardized assessment. Safety monitoring is a mandatory component in all clinical trials (22), including those involving Traditional Chinese Medicine. To ensure consistent evaluation, the TCM-COS-RA includes adverse events as a core outcome domain (23).

The internationally recognized evaluation metrics for RA, such as the DAS28 endorsed by ACR/EULAR and traditional disease activity assessment systems, generally have the limitation of single-dimensional evaluation (24). Their core frameworks mainly rely on objective Western medicine parameters such as joint mobility and inflammatory indicators, without integrating patient-reported outcomes and TCM syndrome differentiation into the standardized assessment system. Take the clinical decision tree proposed by the French Society of Rheumatology in 2004 as an example (25), its assessment system includes three objective indicators: disease activity (DAS28), rheumatoid factor status, and structural damage. By contrast, the RA-TCM-COS retains the quantitative rigour of evidence-based measures while adding patient-reported outcome scales to capture subjective experience. It adopts an integrated model of Chinese and Western medicine that prioritises TCM syndrome evaluation. This represents a shift from single-pathology indicators to a multidimensional assessment.

The RA-COS multidimensional assessment employs an integrated framework combining holistic and localised approaches alongside objective and subjective measures. It encompasses five key domains: comprehensive disease evaluation, physicochemical indicators, quality of life, Traditional Chinese Medicine syndromes, and adverse events. This standardised multidimensional assessment system not only comprehensively addresses clinical requirements for RA disease evaluation but also holds significant value for clinical implementation and application.

Research on cell membrane-mimetic nanoparticles for targeted anti-inflammatory drug delivery centres its core evaluation on inhibiting inflammatory pathways by enhancing delivery efficacy and safety (26). Jo et al. (27) systematic review of 415 randomised controlled trials demonstrated that East Asian herbal medicine with conventional medicine combination therapy (EACM) not only significantly reduced inflammation levels but specifically monitored gastrointestinal damage and hepatic burden. Liu et al.’s (28) research demonstrated that Wutou decoction combined with Western medicine significantly reduced inflammatory markers with a lower incidence of adverse reactions than controls, while proposing combination strategies to mitigate Aconitine risks. A self-assembled clematine-glycyrrhizic acid hydrogel modulated inflammatory pathways and protected osteochondral tissue while monitoring hepatic and renal function (29). These findings correspond to the chemical indicator and safety monitoring domains of the COS. Concurrently, the EACM meta-analysis adopted DAS28 as a secondary key outcome. Studies of aconite decoction reported improvements in both DAS28 and pain scores, aligning with the COS’s emphasis on disease activity and patient-reported outcomes. This provides a unified benchmark for diverse intervention pathways. Moreover, nano-bionic research explicitly targets improved treatment outcomes and quality of life (QoL). This emphasis on patient-reported outcomes and quality of life aligns with RA-TCM-COS’s philosophy of incorporating patient quality of life. Collectively, this evidence underscores the core requirement for comprehensive RA outcome assessment to balance efficacy and safety, alongside objective and subjective measures–precisely the core value of COS-TCM-RA. Current RA-related research often focuses on single dimensions or localised indicators; the COS indicator set holds promise for achieving comprehensive and precise clinical evaluation of RA.

In this study, the TCM syndrome was assessed using a specialized “Syndrome Effectiveness Evaluation Scale” for RA. It is important to note that this scale was developed and preliminarily validated by our research team in a prior methodological study (31) based on the eight core TCM syndrome patterns defined in the Chinese guideline for RA (30). In that study, the final scale demonstrated a reliability coefficient of 0.788 and a structural validity coefficient of 0.735. While this provides a solid foundation for its use, we acknowledge that further validation in broader, independent cohorts will be valuable to fully establish it as a standardized tool for TCM outcome assessment in clinical trials. On this basis, future research can carry out multi-centre and large-sample validation studies to further improve TCM-COS-RA. Moreover, it can explore the effects of different TCM treatment protocols, such as TCM compounding, acupuncture, tuina, on the core indexes, so as to provide a richer theoretical basis and practical guidance for the treatment of RA by TCM. Furthermore, strengthen the integration with Western medicine research, and establish a core index set for RA that combines Western and Chinese medicine to comprehensively improve the diagnosis and treatment of the disease.

## Data Availability

The original contributions presented in the study are included in the article/Supplementary material, further inquiries can be directed to the corresponding author.

## Footnotes

^1^ http://www.comet-initiative.org/studies/details/2126

^2^http://www.chictr.org.cn/index.aspx, https://clinicaltrials.gov/
