# Supplementary material for: Developing a core outcome set for clinical trials of traditional Chinese medicine for rheumatoid arthritis
Source: Front Med (Lausanne). 2025 Dec 8;12:1690963. doi: 10.3389/fmed.2025.1690963 (PMC12719495; doi:10.3389/fmed.2025.1690963)
Supplement: Supplementary file 1 [file Data_Sheet_1.docx]

S1 ****Recommendation Degree of Each Outcome by 117 Doctors****

| Outcomes | S | $\bar{x}$ | R/% | K/% | Highly Recommended % | CV | Second Round |
| --- | --- | --- | --- | --- | --- | --- | --- |
| **Overall disease evaluation** |  |  |  |  |  |  |  |
| 28-joint Disease Activity Score (DAS28) | 932 | 7.97 | 3.42 | 44.44 | 88.89 | 0.19 | √ |
| Physician Global Visual Assessment (PhGV) | 904 | 7.73 | 3.42 | 37.61 | 85.47 | 0.20 | √ |
| Effective Ratio | 886 | 7.57 | 2.56 | 41.88 | 80.34 | 0.25 | √ |
| Chinese Patient-Reported Activity Index for Rheumatoid Arthritis (CPRI-RA) | 883 | 7.55 | 2.56 | 34.19 | 80.34 | 0.21 | √ |
| Clinical Disease Activity Index (CDAI) | 874 | 7.47 | 3.42 | 28.21 | 80.34 | 0.22 |  |
| Patient Global Visual Assessment (PGV) | 861 | 7.43 | 1.71 | 28.21 | 80.34 | 0.25 |  |
| ACR20/50/70 Response Rate | 883 | 7.55 | 1.71 | 34.19 | 79.49 | 0.22 |  |
| Simplified Disease Activity Index (SDAI) | 869 | 7.43 | 3.42 | 31.62 | 77.78 | 0.23 |  |
| **Signs and Symptoms** |  |  |  |  |  |  |  |
| Swollen Joint Count (SJC) | 947 | 8.09 | 0.00 | 49.57 | 93.16 | 0.13 | √ |
| Tender Joint Count (TJC) | 936 | 8.00 | 0.85 | 43.59 | 90.60 | 0.14 | √ |
| Morning Stiffness Duration | 896 | 7.66 | 0.85 | 37.61 | 88.03 | 0.21 | √ |
| Pain Visual Analogue Scale (VAS) | 907 | 7.75 | 0.00 | 41.03 | 87.18 | 0.19 | √ |
| Pain Degree Score | 905 | 7.74 | 0.00 | 37.61 | 86.32 | 0.20 |  |
| Swelling Degree Score | 918 | 7.85 | 1.71 | 43.59 | 85.47 | 0.17 |  |
| Tenderness Degree Score | 904 | 7.73 | 0.00 | 39.32 | 84.62 | 0.19 |  |
| Joint Swelling Index | 885 | 7.56 | 1.71 | 34.19 | 83.76 | 0.21 |  |
| Joint Tenderness Index | 889 | 7.60 | 0.00 | 35.90 | 82.91 | 0.20 |  |
| Morning Stiffness Degree Score | 868 | 7.42 | 3.42 | 31.62 | 78.63 | 0.24 |  |
| Pain Numerical Rating Scale (NRS) | 877 | 7.50 | 0.00 | 35.90 | 77.78 | 0.22 |  |
| Joint Mobility/Restriction Degree Score | 875 | 7.48 | 0.85 | 29.91 | 77.78 | 0.21 |  |
| Number of Dysfunctional Joints | 872 | 7.45 | 3.42 | 29.06 | 76.92 | 0.20 |  |
| Knee Joint Mobility | 840 | 7.18 | 7.69 | 26.50 | 71.79 | 0.25 |  |
| WOMAC Stiffness | 817 | 6.98 | 2.56 | 23.08 | 70.94 | 0.29 |  |
| WOMAC Pain | 847 | 7.24 | 2.56 | 28.21 | 70.09 | 0.25 |  |
| Average Grip Strength of Both Hands | 833 | 7.12 | 7.69 | 23.93 | 70.09 | 0.23 |  |
| WOMAC Total Score | 825 | 7.05 | 2.56 | 20.51 | 67.52 | 0.26 |  |
| WOMAC Daily Activity | 818 | 6.99 | 2.56 | 20.51 | 65.81 | 0.26 |  |
| **Physical and chemical indicators** |  |  |  |  |  |  |  |
| C-reactive Protein (CRP) | 946 | 8.09 | 2.56 | 51.28 | 91.45 | 0.16 | √ |
| Erythrocyte Sedimentation Rate (ESR) | 927 | 7.92 | 0.85 | 47.01 | 88.89 | 0.16 | √ |
| Anticyclic Citrullinated Peptide Antibody (ACCP) | 903 | 7.72 | 1.71 | 46.15 | 81.20 | 0.22 | √ |
| Rheumatoid Factor (RF) | 890 | 7.61 | 1.71 | 39.32 | 81.20 | 0.23 | √ |
| Antimutated Citrullinated Vimentin Antibody (MCV) | 755 | 6.45 | 5.98 | 17.09 | 64.10 | 0.37 |  |
| Tumor Necrosis Factor-α (TNF-α) | 734 | 6.27 | 7.69 | 11.97 | 53.85 | 0.35 |  |
| **Imaging Evaluation** |  |  |  |  |  |  |  |
| X-ray: Modified Sharp Score | 851 | 7.27 | 2.56 | 29.91 | 78.63 | 0.25 | √ |
| Ultrasound: Szkudlarek M Standard | 826 | 7.06 | 4.27 | 28.21 | 70.09 | 0.29 | √ |
| MRI: RAMRIS Score | 832 | 7.11 | 3.42 | 28.21 | 69.23 | 0.26 |  |
| Ultrasound: Walther M Standard | 826 | 7.06 | 5.13 | 28.21 | 69.23 | 0.28 |  |
| Ultrasound: Semiquantitative Joint Effusion | 793 | 6.78 | 4.27 | 24.79 | 64.96 | 0.33 |  |
| Ultrasound: US7 Semiquantitative Score | 773 | 6.61 | 4.27 | 21.37 | 62.39 | 0.34 |  |
| **quality of life** |  |  |  |  |  |  |  |
| Health Assessment Questionnaire-Disability Index (HAQ-DI) | 878 | 7.50 | 2.56 | 33.33 | 77.78 | 0.21 | √ |
| Short Form 36 (SF-36) | 866 | 7.40 | 2.56 | 32.48 | 74.36 | 0.22 | √ |
| Rheumatoid Arthritis Quality of Life Scale (RAQOL) | 822 | 7.03 | 5.98 | 22.22 | 68.38 | 0.26 |  |
| Quality of Life Inventory-74 (GQOLI-74) | 803 | 6.86 | 0.85 | 23.08 | 64.96 | 0.30 |  |
| **TCM-syndromes** |  |  |  |  |  |  |  |
| TCM Syndrome Score (Chinese New Drug Criteria) | 894 | 7.64 | 1.71 | 0.3419 | 0.8462 | 0.21 | √ |
| TCM Syndrome Efficacy Evaluation Scale | 880 | 7.52 | 2.56 | 0.3333 | 0.8291 | 0.25 | √ |
| **Adverse event rate** |  |  |  |  |  |  |  |
| Liver Function | 913 | 7.80 | 2.56 | 52.14 | 88.03 | 0.25 | √ |
| Blood Routine | 913 | 7.80 | 3.42 | 49.57 | 88.03 | 0.25 | √ |
| Renal Function | 901 | 7.70 | 2.56 | 52.14 | 85.47 | 0.28 | √ |
| Adverse Event Incidence | 827 | 7.07 | 5.13 | 35.04 | 77.78 | 0.34 | √ |
| Vital Signs (Including ECG) | 820 | 7.01 | 427 | 36.75 | 75.21 | 0.36 |  |
| Urinalysis | 802 | 6.85 | 7.69 | 36.75 | 70.94 | 0.39 |  |
| Chest X-ray | 798 | 6.82 | 6.84 | 27.35 | 70.09 | 0.33 |  |

S1 presents the results of the first Delphi round, in which 117 clinicians assessed the importance of 52 outcome indicators using a 9-point Likert scale (1–3: not important; 4–6: important but not critical; 7–9: critical). The expert responses were analyzed using a set of descriptive statistics: the Rank Sum (S), Mean Score ($\bar{\text{x}}$), the percentage of ratings in the Not Important range (R, scores 1–3), the percentage in the Critical range (scores 7–9), the Full-Mark Ratio (K, percentage of maximum 9 score), and the Coefficient of Variation (CV). The pre-defined consensus criteria guided the decision-making process: an indicator was retained for subsequent rounds if ≥70% of ratings fell within the 7–9 range and <15% were within the 1–3 range. Conversely, an indicator was designated for exclusion if ≥70% of ratings were in the 1–3 range and <15% were in the 7–9 range. The mean score ($\bar{x}$) served as a measure of the perceived importance, with $\bar{x}$ ≥ 7.0 indicating a critically important outcome. The Coefficient of Variation (CV) functioned as an inverse proxy for the degree of consensus, where lower values denoted higher agreement among the experts.

S2 **Recommendation Degree of Each Outcome Indicator by 40 Experts**

| Outcome Indicators | S | $\bar{x}$ | R/% | K/% | Highly Recommended % | CV |
| --- | --- | --- | --- | --- | --- | --- |
| **Overall disease evaluation** |  |  |  |  |  |  |
| 28-joint Disease Activity Score (DAS28) | 348 | 8.70 | 0.00 | 75.00 | 100.00 | 0.07 |
| Physician Global Visual Assessment (PhGV) | 327 | 8.18 | 0.00 | 52.50 | 90.00 | 0.14 |
| Chinese Patient-Reported Activity Index for Rheumatoid Arthritis (CPRI-RA) | 313 | 7.83 | 0.00 | 37.50 | 90.00 | 0.16 |
| Effective Ratio | 296 | 7.40 | 7.50 | 40.00 | 75.00 | 0.26 |
| **Signs and Symptoms** |  |  |  |  |  |  |
| Swollen Joint Count (SJC) | 345 | 8.63 | 0.00 | 70.00 | 100.00 | 0.07 |
| Tender Joint Count (TJC) | 341 | 8.53 | 0.00 | 65.00 | 97.50 | 0.09 |
| Pain Visual Analogue Scale (VAS) | 327 | 8.18 | 0.00 | 50.00 | 95.00 | 0.12 |
| Morning Stiffness Duration | 333 | 8.32 | 0.00 | 57.50 | 90.00 | 0.12 |
| **Physical and chemical indicators** |  |  |  |  |  |  |
| C-reactive Protein (CRP) | 341 | 8.53 | 0.00 | 67.50 | 97.50 | 0.10 |
| Erythrocyte Sedimentation Rate (ESR) | 335 | 8.37 | 0.00 | 60.00 | 95.00 | 0.11 |
| Anticyclic Citrullinated Peptide Antibody (ACCP) | 328 | 8.20 | 0.00 | 52.50 | 92.50 | 0.12 |
| Rheumatoid Factor (RF) | 319 | 7.98 | 0.00 | 47.50 | 87.50 | 0.16 |
| **Imaging Evaluation** |  |  |  |  |  |  |
| X-ray: Modified Sharp Score | 308 | 7.70 | 0.00 | 27.50 | 82.50 | 0.15 |
| Ultrasound: Szkudlarek M Standard | 295 | 7.38 | 0.00 | 22.50 | 72.50 | 0.18 |
| **quality of life** |  |  |  |  |  |  |
| Health Assessment Questionnaire-Disability Index (HAQ-DI) | 316 | 7.90 | 0.00 | 37.50 | 92.50 | 0.14 |
| Short Form 36 (SF-36) | 309 | 7.73 | 0.00 | 35.00 | 87.50 | 0.17 |
| **TCM-syndromes** |  |  |  |  |  |  |
| TCM Syndrome Score (Chinese New Drug Criteria) | 320 | 8.00 | 0.00 | 42.50 | 95.00 | 0.14 |
| TCM Syndrome Efficacy Evaluation Scale | 316 | 7.90 | 0.00 | 35.00 | 92.50 | 0.15 |
| **Adverse event rate** |  |  |  |  |  |  |
| Liver Function | 331 | 8.28 | 0.00 | 52.50 | 95.00 | 0.11 |
| Blood Routine | 327 | 8.18 | 0.00 | 50.00 | 95.00 | 0.13 |
| Renal Function | 328 | 8.20 | 2.50 | 55.00 | 92.50 | 0.15 |
| Adverse Event Incidence | 317 | 7.93 | 2.50 | 45.00 | 87.50 | 0.18 |

The 22 outcomes retained from the first Delphi round were evaluated in a second round by a panel of 40 senior experts. As presented in S2, the results demonstrated a strong and unanimous consensus, characterized by markedly high mean scores ($\bar{\text{x}}$), Highly Recommended percentages, and K values, concurrently with minimal R values and low coefficients of variation (CV) across all indicators. This convergence of opinion affirmed the critical importance of the candidate outcomes. Consequently, as no further refinement was achievable through statistical analysis alone, the results were advanced to a consensus meeting for the final selection of the core outcome set.

S3 **Recommendation Degree of Each Outcome Indicator by 38 Experts**

| Outcome Indicators | inconclusive | disagreement | consent | Percentage agreement | Reach a consensus |
| --- | --- | --- | --- | --- | --- |
| **Overall disease evaluation** |  |  |  |  |  |
| 28-joint Disease Activity Score (DAS28) | 0 | 0 | 38 | 100.00 | √ |
| Chinese Patient-Reported Activity Index for Rheumatoid Arthritis (CPRI-RA) | 3 | 0 | 35 | 92.11 | √ |
| **Physical and chemical indicators** |  |  |  |  |  |
| C-reactive Protein (CRP) | 0 | 0 | 38 | 100.00 | √ |
| Erythrocyte Sedimentation Rate (ESR) | 0 | 0 | 38 | 100.00 | √ |
| Ultrasound: Szkudlarek M Standard | 6 | 1 | 31 | 81.58 | √ |
| **quality of life** |  |  |  |  |  |
| Health Assessment Questionnaire-Disability Index (HAQ-DI) | 0 | 0 | 38 | 100.00 | √ |
| **TCM-syndromes** |  |  |  |  |  |
| TCM Syndrome Efficacy Evaluation Scale | 2 | 0 | 36 | 94.74 | √ |
| **Adverse event rate** |  |  |  |  |  |
| Adverse Event Incidence | 1 | 0 | 37 | 97.37 | √ |
| Liver Function | 1 | 0 | 37 | 97.37 | √ |
| Renal Function | 1 | 0 | 37 | 97.37 | √ |
| Blood Routine | 2 | 0 | 36 | 94.74 | √ |

The final Delphi round, conducted with 38 experts, aimed to formally ratify the core outcome set.The results, detailed in S3, demonstrate that all 11 candidate outcomes surpassed this threshold, leading to the formal establishment of the final COS-TCM-RA comprising 11 indicators across five domains.

S4 first round Delphi Expert Panel Participants

| Number | Name | Institution | Title | Service time(years) |
| --- | --- | --- | --- | --- |
| 1 | Haili Cao | Hami Central Hospital | associate chief physician | 20 |
| 2 | Cuiqing Zeng | Hainan Provincial Hospital of Traditional Chinese Medicine | associate chief physician | 22 |
| 3 | Weihong Chao | Nanchang Hongdu Hospital of Traditional Chinese Medicine | chief physician | 38 |
| 4 | Wenhua Chen | Ji 'an Central People's Hospital | Attending physician | 18 |
| 5 | Xiaozhong Chen | Changsha County Hospital of Traditional Chinese Medicine | chief physician | 31 |
| 6 | Yanlin Chen | Yunnan Provincial Hospital of Traditional Chinese Medicine | chief physician | 28 |
| 7 | Shikui Cui | Chongqing Hospital of Traditional Chinese Medicine | chief physician | 27 |
| 8 | Weizhe Deng | 962 Hospital, Harbin City, Heilongjiang Province | associate chief physician | 25 |
| 9 | Qiumei Dong | Inner Mongolia Medical University Affiliated Mongolian Hospital | chief physician | 35 |
| 10 | Yongfei Fang | First Affiliated Hospital of Army Medical University | chief physician | 39 |
| 11 | Mingli Gao | Liaoning University of Traditional Chinese Medicine Affiliated Hospital | chief physician | 39 |
| 12 | Lin Ge | Guanganmen Hospital, China Academy of Chinese Medical Sciences | chief physician | 22 |
| 13 | Gang Guo | Hebei Yiling Hospital | chief physician | 32 |
| 14 | Jian Guo | Nanchang University First Affiliated Hospital | chief physician | 32 |
| 15 | Xiaohui Han | Changchun Hengkang Hospital of Traditional Chinese Medicine | Attending physician | 15 |
| 16 | Dongyi He | Shanghai Guanghua Integrated Traditional Chinese and Western Medicine Hospital | chief physician | 33 |
| 17 | Xiaoxiu He | Guanganmen Hospital | chief physician | 33 |
| 18 | Hongjun Huang | Shulan People's Hospital | chief physician | 25 |
| 19 | Qingchun Huang | Guangdong Provincial Hospital of Traditional Chinese Medicine | chief physician | 35 |
| 20 | Wei Ji | Jiangsu Provincial Hospital of Traditional Chinese Medicine | chief physician | 35 |
| 21 | Dexun Jiang | Seventh Medical Center of PLA General Hospital | chief physician | 37 |
| 22 | Quan Jiang | Guanganmen Hospital | chief physician | 39 |
| 23 | Xiaofan Jiang | Shaanxi Provincial People's Hospital | chief physician | 14 |
| 24 | Xiaofeng Jiang | Chongqing Dianjiang County Hospital of Traditional Chinese Medicine | Attending physician | 13 |
| 25 | Juan Jiao | Guanganmen Hospital | chief physician | 15 |
| 26 | Weiping Kong | China-Japanese Friendship Hospital | chief physician | 25 |
| 27 | Peng Lei | Shaanxi Provincial People's Hospital | chief physician | 30 |
| 28 | Juan Li | Southern Hospital, Southern Medical University | chief physician | 38 |
| 29 | Manyi Li | Henan Rheumatism Hospital | chief physician | 26 |
| 30 | Qin Li | Qinghai Provincial Hospital of Traditional Chinese Medicine | chief physician | 30 |
| 31 | Weiguo Li | Chongqing Tongliang District Hospital of Traditional Chinese Medicine | chief physician | 30 |
| 32 | Wenyan Li | Red Star Hospital of the 13th Division of the Production Corps | chief physician | 25 |
| 33 | Zeguang Li | First Affiliated Hospital of Heilongjiang University of Traditional Chinese Medicine | chief physician | 37 |
| 34 | Zhaofu Li | Yunnan University of Traditional Chinese Medicine | chief physician | 25 |
| 35 | Zhenbin Li | Bethune International Peace Hospital | chief physician | 33 |
| 36 | Zheng Li | Wangjing Hospital, China Academy of Chinese Medical Sciences | chief physician | 31 |
| 37 | Hongbo Li | The Third Clinical Hospital affiliated to Changchun University of Traditional Chinese Medicine | associate chief physician | 21 |
| 38 | Yi Liang | Sichuan Provincial Orthopedic Hospital | chief physician | 37 |
| 39 | Chuanhui Liu | Zhengzhou Orthopedic Hospital | associate chief physician | 30 |
| 40 | Hongxiao Liu | Guanganmen Hospital, China Academy of Chinese Medical Sciences | chief physician | 20 |
| 41 | Liping Liu | Shanxi Provincial Hospital of Traditional Chinese Medicine | chief physician | 37 |
| 42 | Qiuhong Liu | Suzhou Traditional Chinese Medicine Hospital | chief physician | 32 |
| 43 | Wei Liu | First Affiliated Hospital of Tianjin University of Traditional Chinese Medicine | chief physician | 30 |
| 44 | Ying Liu | Shandong University of Traditional Chinese Medicine Affiliated Hospital | chief physician | 34 |
| 45 | Zhangkai Liu | Wuning County Hospital of Traditional Chinese Medicine | Attending physician | 18 |
| 46 | Xiuyun Lu | Second Affiliated Hospital of Harbin Medical University | associate chief physician | 11 |
| 47 | Cong Ma | Beijing Hospital of Traditional Chinese Medicine, Capital Medical University | associate chief physician | 8 |
| 48 | Guiqin Ma | Guanganmen Hospital | chief physician | 32 |
| 49 | Jili Ma | Siping Hospital of Traditional Chinese Medicine | chief physician | 26 |
| 50 | Wukai Ma | The Second Affiliated Hospital of Guizhou University of Traditional Chinese Medicine | chief physician | 30 |
| 51 | Qingliang Meng | Henan Provincial Hospital of Traditional Chinese Medicine | chief physician | 28 |
| 52 | Lisha Mo | Affiliated Hospital of Jiangxi University of Traditional Chinese Medicine | Attending physician | 10 |
| 53 | Jinghu Niu | Xi 'an Traditional Chinese Medicine Hospital | associate chief physician | 26 |
| 54 | Xuefeng Pang | Guangxi University of Traditional Chinese Medicine Affiliated Ruikang Hospital | chief physician | 32 |
| 55 | Jiangyun Peng | Yunnan Provincial Hospital of Traditional Chinese Medicine | chief physician | 39 |
| 56 | Yan Qi | Beijing Tongrentang Hospital of Traditional Chinese Medicine | chief physician | 40 |
| 57 | Yue Qu | Mehekou Central Hospital | associate chief physician | 26 |
| 58 | Baodi Ren | Xi 'an Fifth Hospital | associate chief physician | 15 |
| 59 | Bin Ren | Daqing Oilfield General Hospital | chief physician | 26 |
| 60 | Hongbo Shen | Peking University Third Hospital | chief physician | 30 |
| 61 | Kang Shen | Zaozhuang Hospital of Traditional Chinese Medicine, Shandong Province | chief physician | 33 |
| 62 | Changjian Sheng | Fengyang County Hospital of Traditional Chinese Medicine | associate chief physician | 25 |
| 63 | Guixiu Shi | First Affiliated Hospital of Xiamen University | chief physician | 35 |
| 64 | Fuxue Song | First Affiliated Hospital of Army Medical University | associate chief physician | 23 |
| 65 | Xinwei Song | Zhejiang Provincial Hospital of Traditional Chinese Medicine | chief physician | 40 |
| 66 | Xuansong Tan | Chongqing Yubei District People's Hospital | associate chief physician | 13 |
| 67 | Xiaohu Tang | Yunnan Provincial Hospital of Traditional Chinese Medicine | chief physician | 30 |
| 68 | Xiaopo Tang | Guangan men Hospital, China Academy of Chinese Medical Sciences | chief physician | 29 |
| 69 | Qingwen Tao | China-Japanese Friendship Hospital | chief physician | 34 |
| 70 | Xueqiu Tian | Jilin Academy of Traditional Chinese Medicine | associate chief physician | 17 |
| 71 | Shenghao Tu | Huazhong University of Science and Technology HUST | chief physician | 30 |
| 72 | Bei Wang | Beijing Hospital of Traditional Chinese Medicine, Capital Medical University | chief physician | 30 |
| 73 | Chengwu Wang | Affiliated Hospital of Changchun University of Traditional Chinese Medicine | chief physician | 36 |
| 74 | Gang Wang | Gansu University of Traditional Chinese Medicine Affiliated Hospital | chief physician | 36 |
| 75 | Haidong Wang | Gansu Provincial Hospital of Traditional Chinese Medicine | chief physician | 38 |
| 76 | Hailong Wang | Dongzhimen Hospital of Beijing University of Chinese Medicine | chief physician | 15 |
| 77 | Jianming Wang | China-Japanese Friendship Hospital | chief physician | 28 |
| 78 | Yijun Wang | Wangjing Hospital, China Academy of Chinese Medical Sciences | chief physician | 35 |
| 79 | Zhenyu Wang | Second Affiliated Hospital of Harbin Medical University | chief physician | 40 |
| 80 | Shufeng Wei | Fangshan Hospital of Traditional Chinese Medicine | chief physician | 27 |
| 81 | Chengping Wen | Zhejiang University of Traditional Chinese Medicine | chief physician | 28 |
| 82 | Jinyu Wu | The First Affiliated Hospital of Guangxi University of Traditional Chinese Medicine | chief physician | 34 |
| 83 | Yang Wu | Yunnan Provincial Hospital of Traditional Chinese Medicine | chief physician | 38 |
| 84 | Jingjing Xie | Shenzhen Hospital of Traditional Chinese Medicine | Attending physician | 8 |
| 85 | Qingbin Xie | Nanchang University Second Affiliated Hospital | associate chief physician | 22 |
| 86 | Yinfang Xie | Chongqing Jiulongpo District Hospital of Traditional Chinese Medicine | associate chief physician | 10 |
| 87 | Yan Xiong | Nanchang Hongdu Hospital of Traditional Chinese Medicine | chief physician | 38 |
| 88 | Penggang Xu | Xi 'an Fifth Hospital | chief physician | 27 |
| 89 | Weidong Xu | Affiliated Hospital of Jiangxi University of Traditional Chinese Medicine | chief physician | 14 |
| 90 | Luan Xue | Shanghai University of Traditional Chinese Medicine Affiliated Yueyang Hospital of Integrated Traditional Chinese and Western Medicine | chief physician | 30 |
| 91 | Faqing Yang | Chongqing Guochang Bi Traditional Chinese Medicine Orthopedic Hospital | Attending physician | 5 |
| 92 | Huiqin Yang | Wuhan First Hospital | chief physician | 32 |
| 93 | Lei Yi | Shaanxi Provincial People's Hospital | chief physician | 27 |
| 94 | Jing Yu | Liaoning University of Traditional Chinese Medicine Affiliated Hospital | chief physician | 22 |
| 95 | Qinghong Yu | Pearl River Hospital, Southern Medical University | chief physician | 33 |
| 96 | Jianping Yu | Affiliated Hospital of Jiangxi University of Traditional Chinese Medicine | chief physician | 37 |
| 97 | Guojian Zhang | Beijing Hospital of Traditional Chinese Medicine, Pinggu Hospital | associate chief physician | 27 |
| 98 | Haibo Zhang | Changchun Hengkang Hospital of Traditional Chinese Medicine | chief physician | 25 |
| 99 | Huadong Zhang | Guanganmen Hospital, China Academy of Chinese Medical Sciences | chief physician | 32 |
| 100 | Jianyong Zhang | Shenzhen Hospital of Traditional Chinese Medicine | chief physician | 34 |
| 101 | Junli Zhang | Xi 'an Fifth Hospital | chief physician | 37 |
| 102 | Ling Zhang | Yunnan Provincial Hospital of Traditional Chinese Medicine | Attending physician | 5 |
| 103 | Qin Zhang | Beijing Hospital of Traditional Chinese Medicine, Capital Medical University | chief physician | 25 |
| 104 | Shijun Zhang | Sichuan Second Hospital of Traditional Chinese Medicine | chief physician | 37 |
| 105 | Xiurong Zhang | Fourth Affiliated Hospital of Heilongjiang University of Traditional Chinese Medicine | associate chief physician | 18 |
| 106 | Yingze Zhang | China-Japanese Friendship Hospital | chief physician | 37 |
| 107 | Hengli Zhao | Yantai Hospital of Traditional Chinese Medicine, Shandong Province | chief physician | 31 |
| 108 | Zhongwen Zhao | Fujian Provincial Second People's Hospital | chief physician | 30 |
| 109 | Rige Tu Zhao | Xinjiang Autonomous Region Hospital of Traditional Chinese Medicine | chief physician | 30 |
| 110 | Fuzeng Zheng | Henan Provincial Hospital of Traditional Chinese Medicine | chief physician | 37 |
| 111 | Shengli Zhou | Hefei Second People's Hospital | associate chief physician | 32 |
| 112 | Xiaoli Zhou | Shanxi Provincial Hospital of Traditional Chinese Medicine | chief physician | 27 |
| 113 | Xueping Zhou | Nanjing University of Traditional Chinese Medicine | chief physician | 40 |
| 114 | Zushan Zhou | Honghu City Hospital of Traditional Chinese Medicine, Hubei Province | chief physician | 40 |
| 115 | Wanhua Zhu | Nantong Liangchun Hospital of Traditional Chinese Medicine | chief physician | 50 |
| 116 | Yuelan Zhu | Rheumatology Department, Oriental Hospital, Beijing University of Chinese Medicine | chief physician | 40 |
| 117 | Desheng Zou | Chongqing Jiulongpo District People's Hospital | associate chief physician | 15 |

The first round Delphi Expert Panel comprised rheumatologists from 86 clinical centers across all seven geographic regions of China (North, Northeast, East, Central, South, Southwest, and Northwest), representing 29 provincial-level administrative divisions (provinces, municipalities, and autonomous regions. All experts were selected based on their high professional qualifications, with 5~40 years of clinical experience in the field of rheumatology.

S5 Second round Delphi Expert Panel Participants

| Number | Name | Institutions | Title | Service time(years) |
| --- | --- | --- | --- | --- |
| 1 | Zhongwen Zhao | Second Affiliated People's Hospital of Fujian University of Traditional Chinese Medicine | Chief Physician | 30 |
| 2 | Liping Liu | Shanxi Provincial Hospital of Traditional Chinese Medicine | Chief Physician | 36 |
| 3 | Zhenbin Li | Bethune International Peace Hospital | Chief Physician | 35 |
| 4 | Wu Kai Ma | The Second Affiliated Hospital of Guizhou University of Traditional Chinese Medicine | Chief Physician | 31 |
| 5 | Jianyong Zhang | Shenzhen Hospital of Traditional Chinese Medicine | Chief Physician | 34 |
| 6 | Yuelan Zhu | Rheumatology Department, Oriental Hospital, Beijing University of Chinese Medicine | Chief Physician | 41 |
| 7 | Yi Liang | Rheumatology Department, Sichuan Provincial Orthopedic Hospital | Chief Physician | 37 |
| 8 | Junli Zhang | Xi 'an Fifth Hospital | Chief Physician | 37 |
| 9 | Yan Qi | Beijing Tongrentang Hospital of Traditional Chinese Medicine | Chief Physician | 40 |
| 10 | Bei Wang | Beijing Hospital of Traditional Chinese Medicine, Capital Medical University | Chief Physician | 30 |
| 11 | Zeguang Li | First Affiliated Hospital of Heilongjiang University of Traditional Chinese Medicine | Deputy Chief Physician | 36 |
| 12 | Qingwen Tao | China-Japanese Friendship Hospital | Chief Physician | 34 |
| 13 | Jinyu Wu | The First Affiliated Hospital of Guangxi University of Traditional Chinese Medicine | Chief Physician | 35 |
| 14 | Gang Guo | Hebei Yiling Hospital | Chief Physician | 32 |
| 15 | Zhaori Geritu | Xinjiang Medical University Affiliated Hospital of Traditional Chinese Medicine | Chief Physician | 32 |
| 16 | Wei Ji | Jiangsu Provincial Hospital of Traditional Chinese Medicine | Chief Physician | 35 |
| 17 | Haibo Zhang | Changchun Hengkang Hospital of Traditional Chinese Medicine | Chief Physician | 26 |
| 18 | Qin Li | Qinghai Provincial Hospital of Traditional Chinese Medicine | Chief Physician | 30 |
| 19 | Fuzeng Zheng | Henan Provincial Hospital of Traditional Chinese Medicine | Chief Physician | 38 |
| 20 | Hailong Wang | Dongzhimen Hospital of Beijing University of Chinese Medicine | Chief Physician | 16 |
| 21 | Yongfei Fang | First Affiliated Hospital of Army Medical University | Chief Physician | 39 |
| 22 | Jianping Yu | Affiliated Hospital of Jiangxi University of Traditional Chinese Medicine | Chief Physician | 38 |
| 23 | Cuiqing Zeng | Hainan Provincial Hospital of Traditional Chinese Medicine | Deputy Chief Physician | 25 |
| 24 | Juan Jiao | Guanganmen Hospital | Chief Physician | 16 |
| 25 | Haidong Wang | Gansu Provincial Hospital of Traditional Chinese Medicine | Chief Physician | 38 |
| 26 | Dongyi He | Shanghai Guanghua Integrated Traditional Chinese and Western Medicine Hospital | Chief Physician | 34 |
| 27 | Huadong Zhang | Guang'anmen Hospital of China Academy of Chinese Medical Sciences | Chief Physician | 32 |
| 28 | Lusha Yao | First Affiliated Hospital of Hunan University of Chinese Medicine | Deputy Chief Physician | 12 |
| 29 | Zijing Ge | First Affiliated Hospital of Hunan University of Chinese Medicine | Attending Physician | 7 |
| 30 | Yuxiang Xu | First Affiliated Hospital of Hunan University of Chinese Medicine | Deputy Chief Physician | 20 |
| 31 | Yujia Liu | First Affiliated Hospital of Hunan University of Chinese Medicine | Deputy Chief Physician | 9 |
| 32 | Ying Tian | First Affiliated Hospital of Hunan University of Chinese Medicine | Attending Physician | 7 |
| 33 | Xiao Po Tang | Guanganmen Hospital, China Academy of Chinese Medical Sciences | Chief Physician | 29 |
| 34 | Yiying Wu | First Affiliated Hospital of Hunan University of Chinese Medicine | Attending Physician | 10 |
| 35 | Xiao Su | Shanghai Hospital of Traditional Chinese Medicine | Chief Physician | 38 |
| 36 | Dan Liu | First Affiliated Hospital of Hunan University of Chinese Medicine | Deputy Chief Physician | 10 |
| 37 | Quan Jiang | Guanganmen Hospital | Chief Physician | 40 |
| 38 | Qian Xu | First Affiliated Hospital of Hunan University of Chinese Medicine | Attending Physician | 7 |
| 39 | Qiumei Dong | Inner Mongolia Medical University Affiliated Mongolian Hospital | Chief Physician | 35 |
| 40 | Mingli Gao | Liaoning University of Traditional Chinese Medicine Affiliated Hospital | Chief Physician | 40 |

The Second round Delphi Expert for this second round of the Delphi survey comprised 40 specialists, all holding the title of Associate Chief Physician or above (the vast majority being Chief Physicians). They were drawn from 31 high-calibre hospitals across 19 provinces, municipalities, and autonomous regions nationwide.

S6 Third round Delphi Expert Panel Participants

| Number | Name | Institutions | Title | Service time(years) |
| --- | --- | --- | --- | --- |
| 1 | Xiao Su | Shanghai Hospital of Traditional Chinese Medicine | chief physician | 38 |
| 2 | Huadong Zhang | Guanganmen Hospital | chief physician | 32 |
| 3 | Zushan Zhou | Honghu City Hospital of Traditional Chinese Medicine, Hubei Province | chief physician | 40 |
| 4 | Hailong Wang | Dongzhimen Hospital of Beijing University of Chinese Medicine | chief physician | 16 |
| 5 | Zeguang Li | First Affiliated Hospital of Heilongjiang University of Traditional Chinese Medicine | chief physician | 36 |
| 6 | Xiaopo Tang | Guanganmen Hospital | chief physician | 29 |
| 7 | Jian Liu | The First Affiliated Hospital of Anhui University of Chinese Medicine | chief physician |  |
| 8 | Yan Qi | Beijing Tongrentang Hospital of Traditional Chinese Medicine | chief physician | 40 |
| 9 | Rige Tu Zha | Xinjiang Medical University Affiliated Hospital of Traditional Chinese Medicine | chief physician | 32 |
| 10 | Dongyi He | Shanghai Guanghua Integrated Traditional Chinese and Western Medicine Hospital | chief physician | 34 |
| 11 | Fuzeng Zheng | Henan Provincial Hospital of Traditional Chinese Medicine | chief physician | 38 |
| 12 | Jinyu Wu | The First Affiliated Hospital of Guangxi University of Traditional Chinese Medicine | chief physician | 35 |
| 13 | Mingli Gao | Liaoning University of Traditional Chinese Medicine Affiliated Hospital | chief physician | 40 |
| 14 | Bei Wang | Beijing Hospital of Traditional Chinese Medicine, Capital Medical University | chief physician | 30 |
| 15 | Jing Yu | Liaoning University of Traditional Chinese Medicine Affiliated Hospital | chief physician | 22 |
| 16 | Jiwei | Jiangsu Provincial Hospital of Traditional Chinese Medicine | chief physician | 35 |
| 17 | Quan Jiang | Guanganmen Hospital | chief physician | 40 |
| 18 | Jiangyun Peng | Yunnan Provincial Hospital of Traditional Chinese Medicine | chief physician |  |
| 19 | Yongfei Fang | First Affiliated Hospital of Army Medical University | chief physician | 39 |
| 20 | Jianping Yu | Affiliated Hospital of Jiangxi University of Traditional Chinese Medicine | chief physician | 38 |
| 21 | Wei Liu | First Affiliated Hospital of Tianjin University of Traditional Chinese Medicine | chief physician | 30 |
| 22 | Cuiqing Zeng | Hainan Provincial Hospital of Traditional Chinese Medicine | chief physician | 25 |
| 23 | Yuelan Zhu | Rheumatology Department, Oriental Hospital, Beijing University of Chinese Medicine | chief physician | 41 |
| 24 | Qingwen Tao | China-Japanese Friendship Hospital | chief physician | 34 |
| 25 | Zhongwen Zhao | Second Affiliated People's Hospital of Fujian University of Traditional Chinese Medicine | chief physician | 30 |
| 26 | Xinzhi Wang |  | chief physician |  |
| 27 | Junli Zhang | Xi 'an Fifth Hospital | chief physician | 37 |
| 28 | Qin Li | Qinghai Provincial Hospital of Traditional Chinese Medicine | chief physician | 30 |
| 29 | Haidong Wang | Gansu Provincial Hospital of Traditional Chinese Medicine | chief physician | 38 |
| 30 | Liping Liu | Shanxi Provincial Hospital of Traditional Chinese Medicine | chief physician | 36 |
| 31 | Zhenbin Li | Bethune International Peace Hospital | chief physician | 35 |
| 32 | Haibo Zhang | Changchun Hengkang Hospital of Traditional Chinese Medicine | chief physician | 26 |
| 33 | Gang Guo | Hebei Yiling Hospital | chief physician | 32 |
| 34 | Jianyong Zhang | Shenzhen Hospital of Traditional Chinese Medicine | chief physician | 34 |
| 35 | Qiumei Dong | Inner Mongolia Medical University Affiliated Mongolian Hospital | chief physician | 35 |
| 36 | Yi Liang | Rheumatology Department, Sichuan Provincial Orthopedic Hospital | chief physician | 37 |
| 37 | Ying Liu | Shandong University of Traditional Chinese Medicine Affiliated Hospital | chief physician | 34 |
| 38 | Juan Jiao | Guanganmen Hospital | chief physician | 16 |

The Third round Delphi Expert Panel was conducted with a panel of 38 experts to finalize the outcome list.
